# Research on the innovation of medical expenses protection mechanism for rare disease patients in China

**DOI:** 10.3389/fpubh.2026.1847004

**Published:** 2026-09-04

**Authors:** Meijin Song, Yunhua Li

**Affiliations:** Social Security Research Center, School of Politics and Public Administration, Wuhan University, Wuhan, China

**Keywords:** rare diseases, medical expenses, medical expenses protection mechanism, multi-entity collaboration, mechanism innovation

## Abstract

**Background:**

As China works to establish a mature medical security system by 2030, the heavy medical expenses faced by patients with rare diseases and the inadequacies in the existing protection mechanisms have become increasingly prominent and problematic, creating an urgent need for further research and policy solutions.

**Objectives and methods:**

This study used an explanatory sequential design. First, a questionnaire survey was conducted to investigate the status of the protection provided to patients by the three major security channels (government, market, and social organizations) and to assess the medical burden on patients. Then, interviews were conducted to identify the shortcomings of the three current major security channels, with the goal of developing an innovative mechanism for addressing the medical expenses of patients with rare diseases.

**Results:**

The results from the 720 questionnaires showed that, among rare disease patients who could be contacted through patient organizations and related networks, the three major protection channels currently available are still insufficient to cover their high medical expenses, resulting in a heavy financial burden. The 14 interviews revealed two types of deficiencies in the three channels. First, each channel demonstrated limited protection effectiveness. Second, coordination among the three protection channels was lacking.

**Conclusion:**

Policy and mechanism innovations are needed at two levels. First, to improve the effectiveness of the three channels, the government should expand the breadth and depth of coverage and gradually equalize protection levels across regions; the market should lower access thresholds for patients, develop a multi-tiered product supply system, and improve operational risk prevention and control mechanisms, while social organizations should strengthen their capacity to raise charitable resources, establish a sustainable project operation model, and build collaborative networks. Second, at the level of cross-channel coordination, the expense-sharing responsibilities of each entity must be clarified, the types of expenses and target patient populations covered by multiple entities should be better defined, and a multi-entity collaborative reimbursement mechanism should be established. Ultimately, a collaborative, multi-entity mechanism for covering the medical expenses of patients with rare diseases should be established.

## 1 Introduction

Rare diseases, also known as “orphan diseases,” are defined as conditions with extremely low incidence rates. Despite their low incidence, the small number of patients who suffer from rare diseases face a heavy disease burden, particularly in terms of high medical expenses (1). According to the *China Rare Disease Drug Accessibility Report 2019*, 5,810 patients were registered with the Chinese Organization for Rare Disorders (CORD), of whom some spent as much as 80% of their annual household income on disease treatment (2).

To address this issue, the State Council of China issued the *Opinions on Improving the Insurance and Assistance System for Major and Critical Illnesses* in November 2021. This policy proposes “exploring the establishment of a protection mechanism for rare disease drugs by integrating resources from medical insurance, social assistance, charitable support, and other sources to provide comprehensive protection.” It also calls for “supporting the development of commercial health insurance to meet protection needs beyond BMI” (3). However, as of yet, no policy has been issued to address the development of a protection system for rare disease patients’ medical expenses beyond the cost of drugs.

Policy guidance at the national level indicates that multiple entities and resources should be pooled to provide joint protection for patients with rare diseases. Accordingly, local governments have established rare disease protection strategies that integrate multiple entities based on their respective local characteristics. Based on national policies and the current entities providing such protection, the security channels can be divided into three major categories: government, market, and social organization channels. The protection approaches provided by the government channel can be further divided into five types (4): Basic Medical Insurance (BMI), Critical Illness Insurance (CII), Medical Assistance (MA), the Specific Fund for Rare Diseases (SFRD), and the Mutual Medical Fund (MMF). In addition to conventional commercial insurance, the market channel primarily covers rare disease-related expenses for patients through insurance products such as those provided by Huiminbao (City-customized Medical Insurance). As of September 2021, of the 130 Huiminbao products launched, 70 (accounting for 53.8%) provided coverage for rare disease drugs and related medical services (5). In terms of social organizations, several rare disease organizations have been established in China in recent years. In 2021, 130 rare disease organizations carried out regular activities in China (6), providing support to patients with rare diseases through charitable drug donations, financial assistance, and special MA programs (7).

Although various regions of China have implemented preliminary protection measures for rare disease patients, no nationwide protection mechanism has been developed, and insufficient attention has been paid to medical expenses for rare disease patients beyond drug costs. According to the *Outline of the “Healthy China 2030” Plan* issued by the Central Committee of the Communist Party of China and the State Council, China’s healthcare security system should be improved, and the universal healthcare system should become more mature and well-established by 2030 (8). Given the heavy medical expense burden faced by patients with rare diseases, research and innovative policy measures are needed at the national level.

A review of the existing literature shows that current research on rare disease protection mainly concerns drug research and development (9–12), drug supply (13–15), and diagnosis and treatment services (16–19). Thus, scholars have primarily focused on the early research and development stage, the mid-term supply stage, and the diagnosis and treatment stage of the protection system, while discussions of the later stages of protection, particularly protection against long-term medical expense burdens, remain relatively limited. Although a few studies have addressed such later-stage protection (20), they have typically been confined to drug expenses (21–23), with insufficient attention paid to protection mechanisms for other patient expenses. Thus, protection for non-drug expenses has not been adequately explored.

Given this gap, this study focuses on the later stage of the rare disease protection system, with the protection of patients’ overall medical expenditures as its research focus. It aims to address the following research question: “How can protection approaches be improved to alleviate the medical expense burden of patients with rare diseases in China?”

From a theoretical perspective, the protection of medical expenses for rare diseases requires the participation of multiple entities. Moreover, welfare pluralism theory posits that the sources of welfare should be diverse and thus the responsibility for welfare should not be borne solely by the state or the market (24). Adopting this perspective, this study employs an explanatory sequential design to explore innovative approaches to protecting medical expenses for patients with rare diseases in China.

Specifically, a questionnaire survey was used to investigate the composition of the medical expenses incurred by patients with rare diseases in China, analyze the extent of protection provided by various entities, and assess the medical expense burden on patients. Then, semi-structured interviews were conducted with patients with rare diseases or their family members, and the interview data were used to analyze the limitations of current protection approaches. Finally, based on the deficiencies in existing protection approaches, potential policy and mechanism innovations for protecting medical expenses for patients with rare diseases are proposed, with the aim of providing references for future policy formulation.

## 2 Materials and methods

### 2.1 Study design and ethics statement

#### 2.1.1 Study design

An explanatory sequential design was used in this study. First, a questionnaire survey was used to investigate the composition of medical expenses for patients with rare diseases, analyze the current status of reimbursement through the protection methods provided by the three major channels (government, market, and social organizations), and assess the medical expense burden on these patients. Subsequently, interviews were conducted to explore the shortcomings of the current protection methods provided by the three channels in covering the medical expenses of patients with rare diseases.

#### 2.1.2 Ethics statement

Before the questionnaire survey, the researchers introduced the study objectives and content to the participants through the questionnaire link. Participants provided informed consent by clicking the consent button on the webpage. For minor patients or those lacking the capacity to provide independent informed consent, informed consent was provided by their parents or legal guardians through the same procedure, and the questionnaires were completed by their parents or legal guardians on their behalf.

Before the interviews, the researchers introduced the study objectives and content to the participants. Patients who were able to independently complete the interviews provided electronic informed consent themselves. For patients lacking the capacity to provide independent informed consent, proxy informed consent was obtained from their parents or legal guardians. All collected data were anonymized and kept strictly confidential.

According to the relevant provisions of the Measures for Ethical Review of Life Science and Medical Research Involving Human Subjects (2023), this study met the criteria for exemption from ethical review. The study was conducted anonymously, did not collect information that could directly identify participants, did not cause additional harm to participants, and did not involve commercial interests.

### 2.2 Participant recruitment and inclusion criteria

This study received support from several rare disease social organizations: CORD, Pid Kids Care Association, and Hope Star Rare Disease Care Center. Of these, CORD has operated in China for 13 years and has gradually become the largest and most influential rare disease patient organization in the country (25). Research participants for this study were primarily recruited through these rare disease social organizations.

Two inclusion criteria were applied to survey participants: first, a rare disease diagnosis and a diagnosis certificate issued by a physician; second, having incurred medical expenses due to the rare disease. It should be noted that, although China currently defines rare diseases primarily through two rare disease lists, namely *China’s First List of Rare Diseases* (26) and *China’s Second List of Rare Diseases* (27), restricting this study to only those patients with diseases included in these lists would lead to selection bias, as the burden and protection approaches for patients with rare diseases not included in those lists also need to be discussed. Therefore, while this study focuses on patients with rare diseases that are included in the lists, patients with rare diseases not included in the lists were also eligible to participate in the survey. Specifically, patients whose diseases are not included in the lists were included in the survey sample as long as their disease could be found on the official Orphanet website.

### 2.3 Quantitative data collection and analysis

During the design phase, a preliminary draft questionnaire was developed based on the question design of the *2020 China Comprehensive Social Survey on Rare Diseases*, adapted to the specific research objectives of this study. Then, physicians with experience in diagnosing rare diseases, experts from a range of fields (i.e., health economics, sociology, public administration, etc.), as well as staff from rare disease patient organizations, and rare disease patients and their family members were contacted to evaluate the questionnaire items. Revisions were then made based on their feedback.

The questionnaire collection process was conducted in two stages. The first stage was a pilot survey, in which 30 questionnaires were distributed and collected online. Based on feedback from the respondents, the questionnaire was then further revised to clarify items that respondents found confusing, thereby ensuring that all questions were clear and understandable. In the second stage, the final, formal online questionnaire was distributed with the assistance of rare disease patient organizations (see Supplementary material 1) to collect information on the medical expenses incurred by patients with rare diseases during the preceding year. All participants were required to provide consent by clicking the survey link before completing the questionnaire.

During questionnaire collection, a total of 869 questionnaires were returned. After careful review, 149 invalid questionnaires were excluded, yielding 720 valid questionnaires and an effective response rate of 82.8%. Of these, 520 questionnaires were completed by the patients themselves, and the remaining 200 were completed by family members on their behalf. The survey respondents represented 135 types of rare diseases, of which 119 are included in China’s rare disease lists. Of the disease types covered in this survey, rare neurologic diseases accounted for the highest proportion of responses (19.31%), which is consistent with the disease distribution reported in a previous study on the spectrum and epidemiology of rare diseases among a natural population of 14.31 million people in China (28), which found that rare neurologic diseases predominated. The surveyed patients came from 29 provinces, autonomous regions, and municipalities directly under the central government, with a relatively high proportion from East China and North China (58.06%). This finding is consistent with the results of an earlier study that used the National Rare Diseases Registry System to analyze China’s national rare disease population, which found 60.91% of cases to be from regions with higher socioeconomic levels, namely East China and North China (29).

Descriptive analyses of the questionnaire data were conducted using Microsoft Excel 2016 to examine the composition of patients’ medical expenses, the reimbursement status of their medical expenses, and the medical expense burden on patients. The mean reimbursement amounts were calculated based on the entire sample.

### 2.4 Qualitative interview and analysis

The research objective of this study is to analyze the deficiencies of current protection approaches across different channels, and thus the design of the interview guide (see Supplementary material 2) focused on the types of medical protection received by patients with rare diseases and the barriers to accessing such protection that they have encountered. Physicians with experience in diagnosing rare diseases, experts from different fields (i.e., health economics, sociology, public administration, etc.), as well as staff from rare disease patient organizations, and patients and their family members were invited to review and refine the interview questions.

A total of 14 patients with rare diseases were recruited using snowball sampling to participate in semi-structured interviews. The patients varied in age (ranging from 2 to 68 years), family conditions, and sex (35.71% were female). For some patients with limited communication ability (i.e., minors and the older adults), their legal guardians or immediate family members participated in the interviews on their behalf. The interviews were conducted face-to-face, by telephone, or via online video, with an average duration of 30 min to 1 h. The interview sample covered 11 types of rare diseases.

The interview data were analyzed in two stages. In the first stage, the interview materials were transcribed and anonymized through participant coding. Specifically, to protect patient privacy, respondents were coded by disease and individual number, with diseases represented by their English abbreviations and individual numbers beginning from 01 (see Table 1). In the second stage, thematic analysis was conducted using NVivo 11 to assess the limitations of the existing protection approaches provided through different channels to patients with rare diseases. Thematic analysis is a fundamental method of qualitative analysis that can take a range of forms in practice. It does not necessarily aim to generate theory, and it is relatively flexible, as it can be used for both inductive and deductive analysis (30). In this study, two researchers independently coded the interview transcripts. The inter-coder percent agreement was 0.87, and Cohen’s kappa coefficient was 0.82, indicating a high level of agreement between the two researchers. In addition, the interview data from the final participant were used to assess thematic saturation. No new themes were found to have emerged beyond those already identified.

**Table 1:** Rare diseases and codes of interviewed patients.

**Respondent code**	**Province**	**Disease**	**Whether the respondent is the patient**
GSD01	Fujian	Gorham–Stout disease(GSD)	Yes
PMD02	Hebei	Progressive muscular dystrophy (PMD)	Yes
ND03	Guangdong	Norrie disease (ND)	No
MDA504	Hubei	Anti-MDA5 antibody-positive dermatomyositis (MDA5)	Yes
XLA05	Shandong	X-linked agammaglobulinemia (XLA)	Yes
XLA06	Guangdong	X-linked agammaglobulinemia (XLA)	No
PID07	Liaoning	Primary immunodeficiency (PID)	Yes
PID08	Zhejiang	Primary immunodeficiency (PID)	Yes
XLA09	Hebei	X-linked agammaglobulinemia (XLA)	No
ALS10	Shandong	Amyotrophic lateral sclerosis (ALS)	No
X-ALD11	Shanghai	X-linked adrenoleukodystrophy (X-ALD)	Yes
SJIA12	Beijing	Systemic juvenile idiopathic arthritis (SJIA)	Yes
LNS13	Zhejiang	Lesch–Nyhan syndrome (LNS)	No
DMD14	Hubei	Duchenne muscular dystrophy (DMD)	No

## 3 Results

### 3.1 Questionnaire survey results

#### 3.1.1 Composition of patients’ medical expenses

The medical expenses of patients with rare diseases consist of both direct and indirect medical expenses (see Table 2). This study reports both mean and median values to characterize the distribution of medical costs among patients. However, considering that this study aimed to identify the composition of medical expenses for patients with rare diseases, assess the overall medical expense burden on these patients, and further analyze reimbursement provided by different protection entities, rather than merely describe individual-level expenditure patterns, the mean was considered a more appropriate primary summary statistic.

**Table 2:** Composition of direct and indirect medical expenses.

Types of expenses	All surveyed patients			Patients with diseases included in the lists			Patients with diseases not included in the lists		
	Mean expense, CNY (%)	Median, CNY	SD, CNY	Mean expense, CNY (%)	Median, CNY	SD, CNY	Mean expense, CNY (%)	Median, CNY	SD, CNY
Direct medical expenses	101194.45			101575.81			94535.39		
Drugs expenses	35228.33 (34.81%)	5200.00	182721.50	36398.35 (35.83%)	5400.00	187717.50	14797.95 (15.65%)	800.00	26363.01
Surgeries expenses	24986.87 (24.69%)	1800.00	101192.00	25349.41 (24.96%)	2000.00	103696.80	18656.41 (19.73%)	0.00	35804.20
Genetic testing and outsourced examination expenses	8397.36 (8.30%)	4000.00	15390.61	8226.58 (8.10%)	4000.00	15429.37	11379.49 (12.04%)	9500.00	14562.92
In-Hospital examination expenses	14431.88 (14.26%)	4000.00	41045.83	14372.18 (14.15%)	4000.00	41769.10	15474.36 (16.37%)	1000.00	25613.77
Medical consumables expenses	3170.83 (3.13%)	600.00	15139.71	3160.11 (3.11%)	600.00	15474.98	3357.95 (3.55%)	600.00	7178.36
Rehabilitation expenses	8071.48 (7.98%)	0.00	25706.75	8305.09 (8.18%)	0.00	26365.97	3992.31 (4.22%)	0.00	6774.78
Other expenses*	6907.70 (6.83%)	0.00	37026.53	5764.09 (5.67%)	0.00	28027.89	26876.92 (28.43%)	0.00	107004.40
Indirect medical expenses	22484.51			22209.62			27284.55		
Round-trip transportation expenses	4737.34 (21.07%)	2000.00	8641.30	4449.98 (20.04%)	2000.00	8303.35	9755.13 (35.75%)	4000.00	12320.32
Off-site accommodation expenses	4878.30 (21.70%)	1000.00	25655.87	4751.64 (21.39%)	1000.00	26265.56	7089.94 (25.99%)	1500.00	10186.56
Special diets expenses	2462.20 (10.95%)	0.00	19472.16	2538.01 (11.43%)	0.00	19996.69	1138.46 (4.17%)	0.00	4096.25
Outsourced drugs expenses	3163.00 (14.07%)	817.50	10813.63	3187.90 (14.35%)	1000.00	11054.52	2728.21 (10.00%)	0.00	5053.80
Assistive devices expenses	2113.20 (9.40%)	200.00	5672.36	1955.82 (8.81%)	200.00	3558.78	4861.28 (17.82%)	0.00	19338.24
Social interaction expenses	5130.47 (22.82%)	500.00	44910.73	5326.27 (23.98%)	500.00	46154.35	1711.59 (6.27%)	0.00	5548.99
Total	123678.96			123785.43			121819.94		

^*^Other expenses include meal expenses during hospitalization and medical service expenses.

Direct medical expenses are defined as expenditures incurred within medical and health institutions, such as drugs, surgeries, genetic testing and outsourced examination, medical consumables, rehabilitation expenses, etc. The survey revealed that the patients’ average annual direct medical expenses amounted to as high as CNY 101,194.45. Of these, drug expenses accounted for the largest proportion, representing 34.81% of the total.

Indirect medical expenses are disease-related expenditures incurred outside medical institutions, such as round-trip transportation, off-site accommodation, special diets, outsourced drugs, assistive devices, and social interaction expenses. The survey revealed that the average annual indirect medical expenses amounted to CNY 22,484.51, of which round-trip transportation expenses averaged CNY 4,737.34, accounting for 21.07% of the total. Off-site accommodation expenses averaged CNY 4,878.30, accounting for 21.70% of the total. Together, these two items accounted for 42.77% of indirect medical expenses. Overall, the surveyed patients incurred average annual medical expenses of CNY 123,678.96.

Further analysis was conducted by stratifying the sample according to whether the patients’ diseases were included in China’s two national rare disease lists. The average medical expenses of the two groups were then calculated. The results show that the average medical expenses were CNY 123,785.43 for patients with listed diseases, with average direct and indirect medical expenses of CNY 101,575.81 and CNY 22,209.62, respectively. For patients with unlisted diseases, the average medical expenses were CNY 121,819.94, with average direct and indirect medical expenses of CNY 94,535.39 and CNY 27,284.55, respectively.

#### 3.1.2 Financial situation of individuals and households

**Figure 1:**
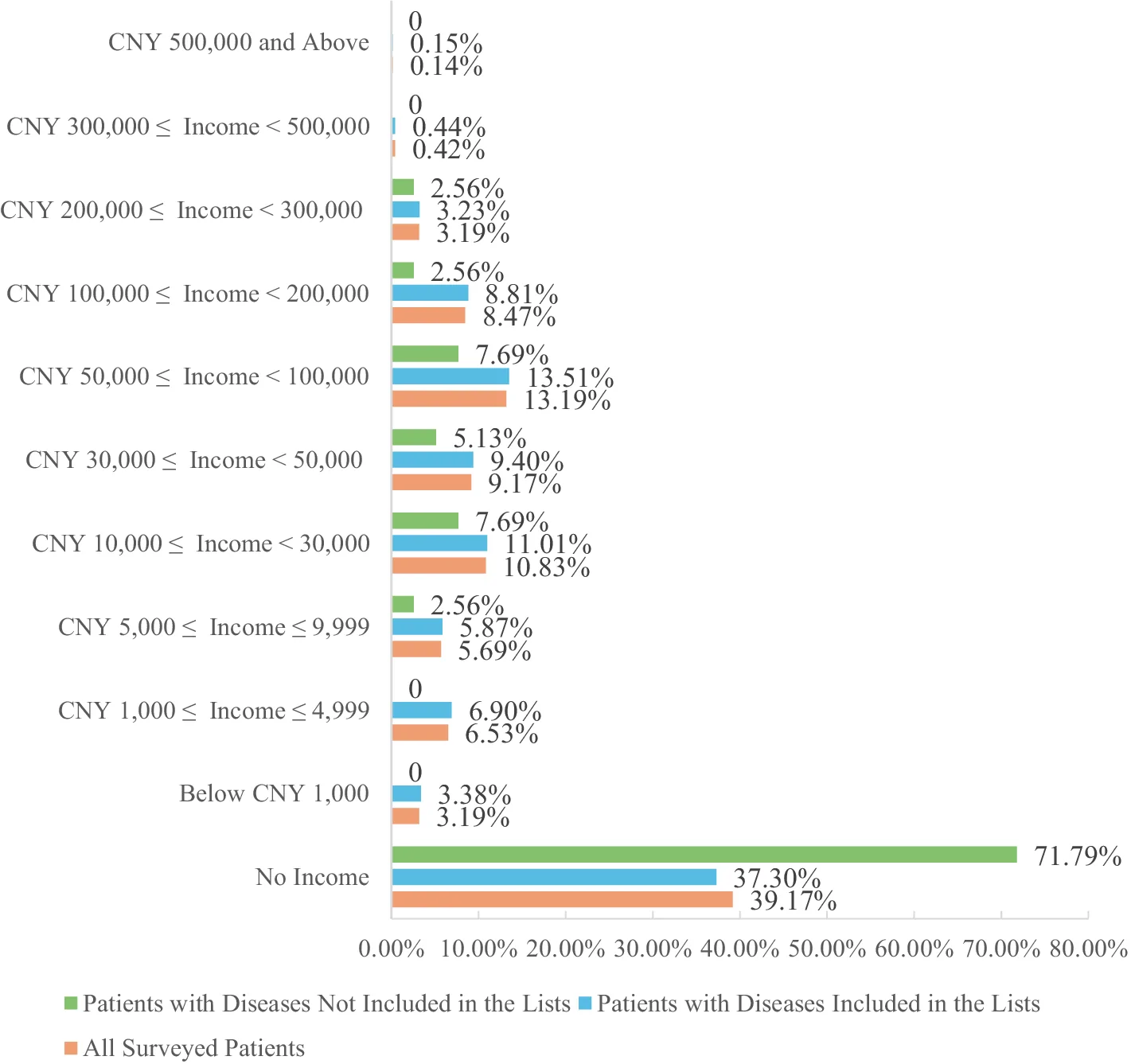
Distribution of individuals’ incomes.

**Figure 2:**
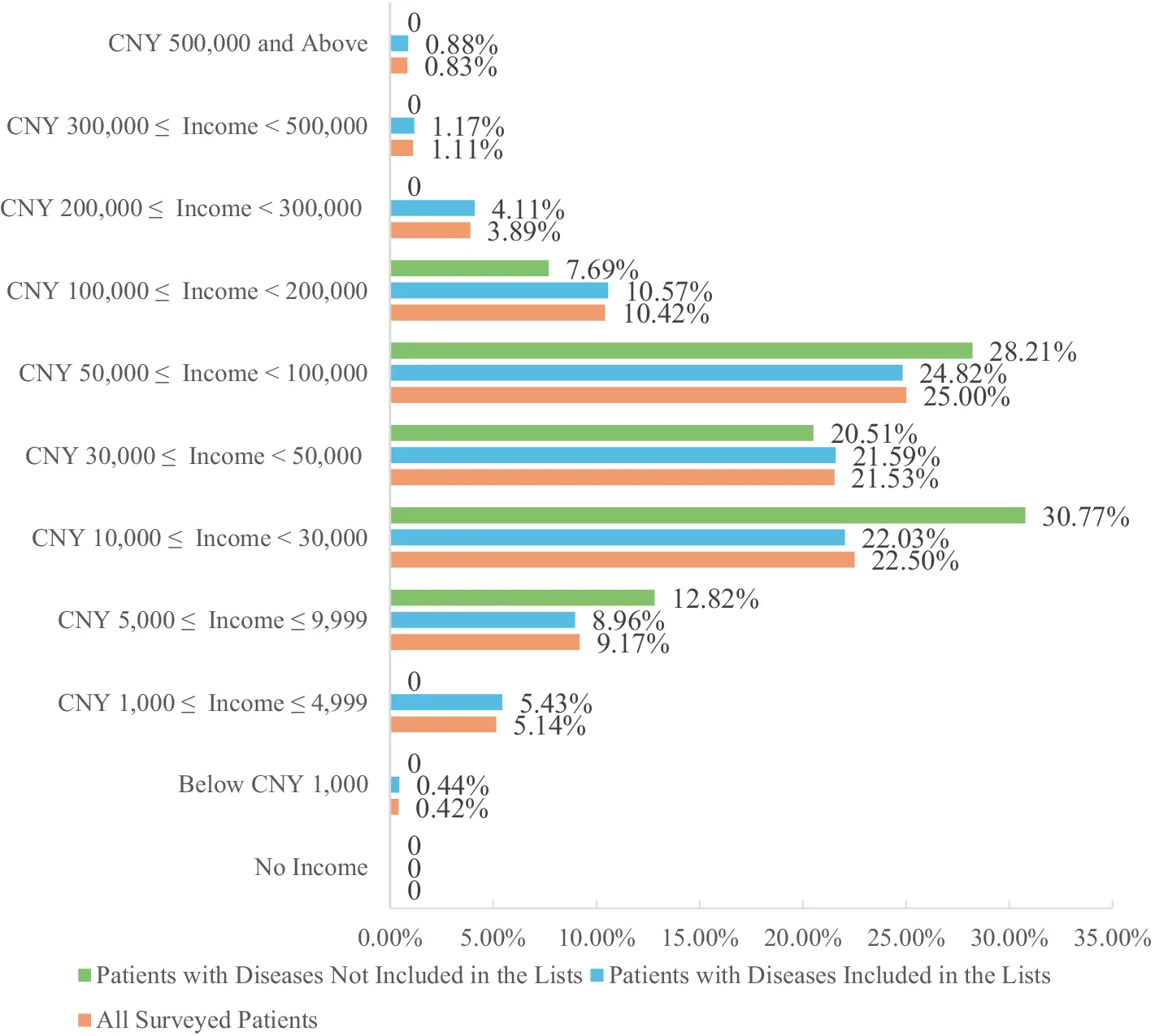
Distribution of household incomes.

The survey data showed that the patients generally had low annual incomes. Only 60.83% of the patients had a source of income (see Figure 1), and the average annual income of those who had an income was CNY 39,478.50. Because the personal income of most patients with rare diseases was insufficient to cover their medical expenses, they had to rely on the income of their household. However, the survey results show that 83.75% of households of patients with rare diseases had an annual income below CNY 100,000 (see Figure 2), with an average annual household income of CNY 68,746.53.

Further analysis was conducted by stratifying the sample according to whether the patients’ diseases were included in China’s two national rare disease lists. The distribution and mean values of annual personal and household income were analyzed among patients with listed vs. unlisted diseases. The results show that, in terms of personal income distribution, 71.79% of patients with unlisted diseases reported no personal income (see Figure 1). For those who had an income, the average annual personal incomes were CNY 40,933.65 and CNY 19,789.50 for patients with listed vs. unlisted diseases, respectively. Regarding household income distribution, both groups were mainly concentrated in the CNY 10,000–50,000 range (see Figure 2). The average annual household incomes were CNY 69,904.10 and CNY 48,012.00 for patients with listed vs. unlisted diseases, respectively.

#### 3.1.3 Reimbursement by various entities

For the reimbursement programs provided through the government channel, the average annual reimbursement amounts received through Basic Medical Insurance, Critical Illness Insurance, the Specific Fund for Rare Diseases, and Medical Assistance were approximately CNY 14,704.99, CNY 13,352.74, CNY 1,434.22, and CNY 2,848.79, respectively. Although the majority of patients (85.42%) received reimbursement through Basic Medical Insurance, their access to the other government-provided reimbursement programs was limited. Only 36.11, 17.50, and 27.08% of patients received reimbursement through Critical Illness Insurance, the Specific Fund for Rare Diseases, and Medical Assistance, respectively.

In comparison, the average amount of financial protection provided through the market channel was relatively low, at approximately CNY 4,125.35, with only 79 patients (10.97% of the surveyed patients) receiving such protection.

The financial protection provided through social organizations was also limited, with an average amount of approximately CNY 2,267.31, insufficient to cover the patients’ high medical expenses. Moreover, only 18.33% of the surveyed patients received such support (see Table 3).

**Table 3:** Average reimbursement amounts for patients with rare diseases across government, market, and social organization channels.

Covered items	All surveyed patients, mean (SD), CNY	Patients with diseases included in the lists, mean (SD), CNY	Patients with diseases not included in the lists, mean (SD), CNY
Government	32448.84	33911.74	6904.11
Basic medical insurance	14704.99 (84059.13)	15271.27 (86358.20)	4816.92 (11601.77)
Critical illness insurance	13352.74 (50424.88)	14053.10 (51747.52)	1123.33 (5389.70)
Specific fund for rare diseases	1434.22 (6594.97)	1516.36 (6772.25)	0.00 (0.00)
Medical assistance	2848.79 (14499.35)	2956.74 (14865.37)	963.85 (4424.29)
Other items*	108.09 (1978.45)	114.28 (2034.22)	0.00 (0.00)
Market	4125.35	4321.09	707.66
Huiminbao	2975.64 (24110.56)	3109.20 (24767.61)	643.56 (4002.82)
Commercial medical insurance	1149.72 (9326.52)	1211.89 (9586.04)	64.10 (400.32)
Social organization	2267.31 (10948.33)	2045.36 (8848.16)	6142.84 (29167.00)
Total	38841.51	40278.20	13754.60

*Other programs include MMF, student health insurance, and government-funded medical care, among others.

Further analysis was conducted by stratifying the sample according to whether the patients’ diseases were included in China’s two national rare disease lists. Descriptive analyses were performed regarding reimbursement received through different channels by patients with listed vs. unlisted diseases. The results show that the average reimbursement amount received by patients with listed diseases was CNY 40,278.20, while the corresponding amount for patients with unlisted diseases was CNY 13,754.60 (see Table 3).

#### 3.1.4 Medical expense burden on patients

On average, the surveyed patients with rare diseases incurred out-of-pocket medical expenses of CNY 84,837.45, equivalent to 214.90% of their average annual personal income and 123.41% of their average annual household income, indicating a substantial financial burden associated with medical expenses.

Further analysis was conducted by stratifying patients according to whether their diseases were included in China’s two national rare disease lists. The results show that the average out-of-pocket medical expenses were CNY 83,507.23 for patients with listed diseases, accounting for 204.01% of their average annual personal income and 119.46% of their average annual household income.

For patients with unlisted diseases, the average out-of-pocket medical expenses were CNY 108,065.34, accounting for 546.07% of their average annual personal income and 225.08% of their average annual household income (see Table 4).

**Table 4:** Individual and household medical financial burdens of patients with rare diseases.

Patient group	Mean expenses, CNY	Mean reimbursement, CNY	Mean out-of-pocket expenses, CNY	Mean annual income, CNY		Medical financial burden, %	
				Personal	Household	Personal	Household
All surveyed patients	123678.96	38841.51	84837.45	39478.50	68746.53	214.90%	123.41%
Included in the lists	123785.43	40278.20	83507.23	40933.65	69904.10	204.01%	119.46%
Not included in the lists	121819.94	13754.60	108065.34	19789.50	48012.00	546.07%	225.08%

**Figure 3:**
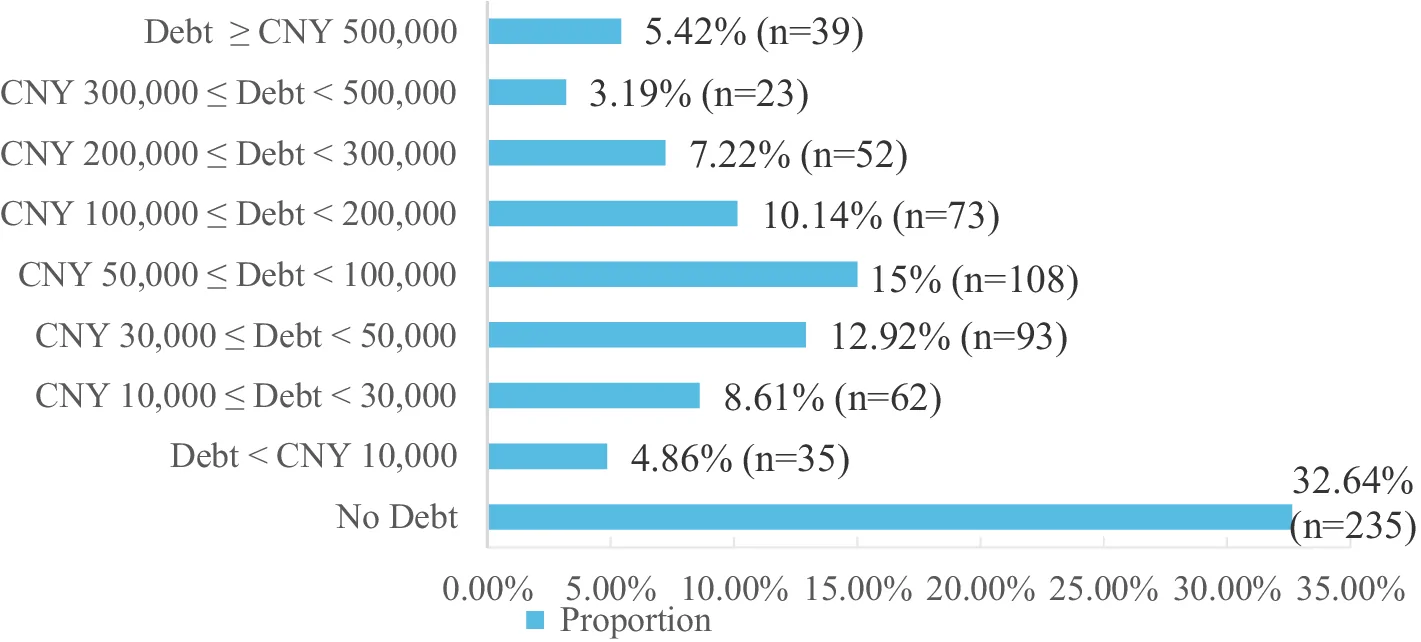
Distribution of household debt among patients with rare diseases.

Unable to bear the substantial financial burden, many families reported having to raise funds through borrowing and other means to sustain the patients’ treatment. Household debt was common among patients with rare diseases: approximately 67.36% of the surveyed households had incurred debt because of high medical expenses, and 5.42% had debts exceeding CNY 500,000 (see Figure 3).

### 3.2 Interview results

After the aforementioned analysis, the deficiencies of current protection approaches were classified into two major categories: “Limited Effectiveness of Protection Approaches across Channels” and “Lack of Coordination across Channels.” The specific deficiencies under each category are described below.

#### 3.2.1 Limited effectiveness of current protection channels

##### 3.2.1.1 Limitations of government protection

1 Limited breadth of coverage

The breadth of coverage is defined here as the scope of medical expenses related to rare diseases that are covered. Constrained by the “Three Catalogues” of China’s National Medical Insurance (i.e., the National Reimbursement Drug List (NRDL), the Medical Consumables Catalogue, and the Diagnosis and Treatment Items Catalogue), protection provided through the government channel is largely limited to patients’ direct medical expenses, with little coverage for their indirect medical expenses. Moreover, even in terms of direct medical expenses, current government protection approaches still exhibit several deficiencies.

The first issue lies in the limited coverage for drug expenses. The most common situation reported by the interviewees is that the drugs they need are not included in the NRDL. As one patient noted, *“The total expenses were more than CNY 300,000.00, but only a few thousand could be reimbursed through insurance. Since some drugs are imported, only a very small portion is covered” (X-ALD11)*. Currently, China’s two national rare disease lists include a total of 207 rare diseases. However, orphan drugs have not yet been approved for marketing in China for all the diseases included in the lists. Moreover, even for orphan drugs that have been so approved, not all are included in the National Reimbursement Drug List (NRDL). By the end of 2025, a total of 140 orphan drugs had been included in the NRDL, covering 71 rare diseases. However, 75 drugs for 61 rare diseases were still not included in the NRDL. For 34 of these rare diseases, none of the available therapeutic drugs were covered. The annual treatment costs for most of these drugs range from several hundred thousand to more than one million CNY, imposing a substantial financial burden on patients and their families (31). Although some provinces and municipalities have established supplementary protection mechanisms for orphan drugs that are not covered by the NRDL, the scope of coverage remains limited. For example, the Specific Fund for Rare Diseases in Zhejiang Province covers only two rare diseases.

Even when a medication is included in the NRDL, reimbursement is often denied because the patient’s condition does not fall within the approved indications for that drug. As one interviewee explained, *“My medication is technically covered under BMI, but since my condition is not among the approved indications, it cannot be reimbursed” (SJIA12).*

In addition to primary treatment drugs, some patients also require adjunct medications that are also excluded from reimbursement. As one patient described, *“Specialists usually prescribe four or five types of supportive medications. Even the cheapest hormones plus these supportive medications cost about CNY 10,000.00 per year, and none of them can be reimbursed” (DMD14).*

Other direct medical expenses, such as surgery, donor matching, and diagnostic testing, are also frequently excluded from reimbursement. One patient explained, *“A hematopoietic stem cell transplant costs nearly CNY 300,000.00, which we simply cannot afford… the matching fees and related expenses are all out-of-pocket, with no reimbursement available” (X-ALD11)*. Another noted, *“I’ve taken the MDA5 antibody test twice, and each test costs about CNY 1,200.00. Tests for bronchial infections cost between CNY 7,000.00 and CNY 8,000.00. None of these are covered by insurance” (MDA504).* Similarly, *“Genetic testing costs more than CNY 4,000.00, and it’s also not reimbursable” (DMD14).*

2 Limited depth of coverage

Depth of coverage is to the extent to which patients’ medical expenses are reimbursed. Although BMI and CII provide relatively high reimbursement rates and ceilings, patients may still find themselves unable to obtain adequate financial protection for a variety of reasons.

First, reimbursement rates may be reduced due to out-of-area healthcare utilization. The uneven regional distribution of healthcare resources for diagnosis and treatment forces many patients with rare diseases to seek medical care outside their home regions. However, some provinces and cities provide relatively low reimbursement rates for such out-of-area healthcare services. For example, in Anhui Province, the reimbursement rate under CII is reduced by 10 percentage points for patients referred for out-of-province treatment or emergency care; it is reduced by 20 percentage points for those seeking care outside their home region without an official referral. As one patient stated, *“We mainly receive treatment in major cities such as Beijing, Shanghai, Guangzhou, and Chongqing… but the reimbursement rate for out-of-province treatment is much lower” (SJIA12).* Furthermore, many patients experience disease flare-ups that are so sudden that they are unable to complete the required referral procedures in time, resulting in further reductions in reimbursement rates. *“Sometimes the situation is urgent, and there’s simply no time to go through those procedures—we can only get hospitalized directly” (PMD02).*

Second, the reimbursement rate under BMI for urban and rural residents is lower than that for urban employees. Many patients with rare diseases are children or adults who are unable to work because of their condition; such patients are typically covered only by the resident insurance scheme. Compared to the employee insurance scheme, the resident insurance scheme provides less comprehensive coverage and lower reimbursement rates. For instance, in Anhui Province, the reimbursement rate under the outpatient program for chronic and special diseases is 60% for individuals covered by the resident insurance scheme, compared with 80% for those covered by the employee insurance scheme. As one respondent remarked, *“My child can only be covered under the standard resident healthcare security scheme, but the reimbursement amount is quite limited” (DMD14).*

3 Substantial regional inequities

Protection inequities mainly take the form of substantial regional disparities in coverage for the same rare disease. As one patient noted, *“In Hubei, our condition is not covered under the outpatient program for chronic and special diseases, but in Anhui and Shandong, it is” (DMD14).* Studies have also shown that patients with the same rare disease may receive different levels of benefit coverage depending on where they live (4). Even when patients attempt to transfer their household registration (hukou) to regions that offer more generous insurance benefits, they are often unable to access those improved benefits immediately. This is because regions that offer stronger protection often impose eligibility restrictions based on local fiscal capacity. For instance, Shandong’s CII, Zhejiang’s SFRD, and Foshan’s MA program typically require patients to participate in the local healthcare security system and maintain a local household registration for at least 5 years before they become eligible for benefits, effectively excluding patients from other regions. In summary, substantial regional disparities in protection have created what patients describe as “different fates for the same disease,” resulting in marked differences in benefit coverage across regions.

##### 3.2.1.2 Limitations of market protection

1 Restrictive eligibility requirements

Conventional commercial insurance products often exclude patients with rare diseases from coverage. Huiminbao, an inclusive insurance product that is guided and supervised by the government but operated by commercial insurers, generally covers the medical expenses of patients with rare diseases. However, it also imposes eligibility restrictions that block some patients from obtaining coverage. Some Huiminbao products exclude genetic diseases from coverage, and many rare diseases are caused by genetic defects. As one participant noted, *“Many insurance plans exclude anything genetic or congenital—they will not cover it” (X-ALD11).* Coverage for patients with pre-existing conditions is often limited as well. *“If you purchased the inclusive insurance before diagnosis, you might get some coverage. But if you buy it after falling ill, it does not work” (X-ALD11).* Many Huiminbao policies explicitly exclude coverage for pre-existing conditions. For example, the terms of Hebei Yanzhao Health Insurance’s basic plan stipulate that, for insured individuals who have any of five specified pre-existing conditions before enrollment, the reimbursement rate for inpatient medical expenses arising from diseases or accidents associated with those conditions is 0% after the policy takes effect. The five categories of pre-existing conditions include rare diseases such as leukemia, hemophilia, and aplastic anemia (32). Consequently, patients who already have these rare diseases may receive little or no financial protection even if they enroll in the insurance plan.

2 Limitations in coverage responsibilities

Most Huiminbao products primarily cover three categories of expenses: out-of-pocket expenses within the scope of medical insurance, out-of-pocket expenses outside the scope of medical insurance, and specialty drug expenses. Specialty drug coverage involves the inclusion of rare disease drugs in a designated specialty drug list, with reimbursement provided at a specified rate.

However, in practice, only limited coverage is provided under these three categories for patients with rare diseases. With respect to in-scope out-of-pocket expenses and out-of-scope out-of-pocket expenses, some Huiminbao products do not cover specific outpatient expenses, nor do they cover outpatient and emergency expenses incurred before or after hospitalization. Patients with rare diseases who do not require hospitalization may therefore receive minimal protection from these products. One interviewee noted,” *I do not need to be hospitalized; I just get my medicine at the clinic… but Huiminbao does not cover that*” *(GSD01).*

With regard to specialty drug coverage, only an extremely limited number of rare disease drugs are included in the Huiminbao formularies. Some products cover fewer than five rare disease drugs, and only a small number cover more than ten. Consequently, such coverage provides little practical benefit for many patients: “*That inclusive insurance’s so-called ‘special drug’ coverage… is completely useless*” *(GSD01)*.

3 Instability in coverage

Huiminbao products are typically issued as one-year policies; patients must renew their coverage annually, and policy terms are frequently revised. Such changes can create challenges for patients with rare diseases, such as loss of eligibility following renewal and difficulties in claim settlement across policy years.

The loss of eligibility can follow renewal when a patient who received reimbursement during one policy year finds, after renewal, that the exclusion clauses have been revised to reclassify their illness as a pre-existing condition, thereby making it ineligible for future reimbursement. This interruption in coverage poses a substantial risk for rare disease patients who require long-term treatment, undermining coverage continuity and reducing confidence in Huiminbao. As one respondent explained, *“In the second year, they would not compensate me again under Huiminbao, so I just stopped renewing it” (XLA09).*

The cross-period claim settlement issue arises when an insured person purchases a Huiminbao plan for 2024 but does not renew it in 2025. If a hospital stay spans two policy years (e.g., admission in 2024 and discharge in 2025), expenses incurred during the latter policy year may not be reimbursed. As one patient noted, *“Since I did not buy it this year, they will not cover me” (XLA09).*

##### 3.2.1.3 Limitations of social organization protection

1 Insufficient financial assistance

Although the number of charitable programs for rare disease patients in China has increased in recent years, the level of assistance they provide remains limited relative to the substantial financial burden those patients face. The amount of assistance provided by social organizations to patients with rare diseases or their households generally ranges from several thousand to tens of thousands of CNY per year. For example, Hemophilia Home provides CNY 2,400.00 per person through its assistance program for financially disadvantaged patients with hemophilia. However, average out-of-pocket medical expenses for such patients amount to approximately CNY 80,000.00. This assistance is thus far from sufficient to alleviate the financial burden faced by patients with rare diseases. As one patient remarked, *“It’s useless; I do not even bother applying for that (charitable aid program) anymore—it’s just too troublesome” (XLA09).*

2 Limited long-term sustainability of support resources

The support provided by social organizations has limited sustainability as well. First, relatively few charitable programs operate on a long-term basis. According to relevant reports, the duration of charitable programs in China has decreased in recent years, with a marked decline in the number of programs lasting 4 years or longer (6). This lack of long-term continuity is even more pronounced for rare disease charities. Although media coverage of specific rare disease cases may temporarily attract public attention and donations, such fundraising typically declines rapidly as public interest wanes, making it difficult to provide sustained support for patients and their families.

Second, even when long-term programs are available, they often restrict eligibility to specific groups in order to contain expenditures. As one interviewee explained, *“This program mainly targets minors; the chances of adults being approved are quite low” (X-ALD11).*

In other cases, programs provide only one-time assistance, making it difficult for patients to receive continued support. *“That assistance program is available every year, but once you have received aid once, the chances of being approved again are very slim” (XLA05).*

3 Uneven distribution of support resources

Some diseases, such as leukemia and hemophilia, have larger patient populations that have facilitated the development of well-established, specialized patient organizations. Most rare disease organizations in China focus on a single disease group, such as the Hemophilia Home Foundation and the Porcelain Dolls Rare Disease Care Center. These organizations generally have stronger organizational capacity, better access to volunteer resources, and the ability to provide disease-specific financial assistance programs. Patients with these conditions can thus seek support not only from comprehensive rare disease charities but also from organizations dedicated to their specific disease.

In contrast, rare diseases that affect smaller patient populations often lack the scale needed to attract sufficient public attention, fundraising resources, and organizational support. For instance, individuals with primary immunodeficiency diseases often have access to only a single source of assistance. *“For our condition, only the Illness Challenge Foundation offers assistance—there’s nothing else. Our volunteers work purely out of love, and we have not received any donations, so we simply cannot provide financial help to our patients” (PID07).* In most cases, organizations established for such conditions are limited to providing only information and emotional support, with little capacity for providing substantial financial assistance.

#### 3.2.2 Lack of coordination across protection channels

##### 3.2.2.1 Overlapping coverage across entities

Currently, multiple entities, including the government, the market, and social organizations, are involved in covering the medical expenses of patients with rare diseases. From a theoretical perspective, these entities should ideally coordinate their resources so that they can perform complementary functions. However, in reality, there is substantial overlap in the types of expenses covered by these entities. Specifically, as noted above, medical expenses for patients with rare diseases can be broadly divided into direct and indirect medical expenses. The government, the market, and social organizations all primarily focus on direct medical expenses, while severely neglecting the need for coverage for indirect medical expenses. As one respondent noted, *“We usually have to travel to other cities for treatment, and transportation expenses are high. Some of our children are in critical condition and need to take ambulances. In some remote areas, there’s no high-speed rail, so we have to fly—which costs even more. None of these expenses are reimbursable” (PID07).*

From the perspective of population coverage, the protection provided through the government channel is primarily available only to patients whose diseases and medications are included in the “Three Catalogues” of China’s National Medical Insurance. Meanwhile, for patients whose diseases or medications fall outside the “Three Catalogues,” or whose conditions have not been included in China’s First or Second List of Rare Diseases, available protection is severely limited. Although Huiminbao provides specialty drug coverage that offers some protection for patients whose medications are not covered by the NRDL, it offers only limited support for those patients whose conditions lack effective drug therapies and who must therefore rely on special medical foods for treatment. *“We need a type of special dietary supplement to slow disease progression—it costs over CNY 4,000.00 per month, about CNY 50,000.00 a year. It’s really unaffordable” (X-ALD11).* Similarly, social organizations tend to focus on rare diseases that are included in the official disease lists, leaving patients with conditions outside these lists with limited access to financial assistance programs.

##### 3.2.2.2 Lack of cross-entity coordination standards

In addition to the problem of overlapping coverage, there is also a lack of effective coordination of the reimbursement mechanisms among the government, market, and social organization channels. The reimbursement systems of these three channels operate independently, without a unified, one-stop reimbursement process. As a result, patients with rare diseases must identify and meet documentation requirements for each channel separately, repeatedly visit medical institutions to obtain the necessary paperwork, and submit reimbursement applications through multiple systems. In the absence of any standardized procedures, this fragmented process can prevent patients who are already physically vulnerable from being able to successfully obtain reimbursement. *“I have rural healthcare security and a few other kinds of insurance, but the reimbursement procedures are so complicated, and each one is different. I just gave up on some of them—I could not handle it physically” (PMD02).*

Some programs also impose stringent documentation requirements that can prevent patients from obtaining reimbursement. *“There’s a program I could apply to for reimbursement, but it requires a low-income certificate, minimum living allowance certificate, and proof of extreme hardship. It also strictly requires that the invoices come from designated hospitals in specific provinces. For people like us, it’s impossible to get reimbursed” (DMD14).*

Moreover, some patients experience acute disease episodes or require urgent hospitalization and interprovincial transfer because their locally available healthcare resources are insufficient. Under such circumstances, patients may lose their reimbursement eligibility because they are unable to prepare the required documentation or complete the required referral procedures in time. *“Sometimes the situation is urgent, and there’s no time to handle those procedures—we just go straight to the hospital” (PMD02).*

The lack of coordination across entities may be due to the fact that different departments, in an effort to protect data privacy and prevent data breaches, have established separate reimbursement requirements and procedures. There is no well-established central data-sharing platform to facilitate information exchange across departments and support the development of standardized reimbursement procedures.

## 4 Discussion

### 4.1 Policy and mechanism innovation for expense protection for rare disease patients

#### 4.1.1 Improving the effectiveness of current protection channels

##### 4.1.1.1 Strengthening the government’s primary protection role

1 Expanding coverage scope

The findings of this study indicate that patients with rare diseases face insufficient protection against medication costs. Therefore, to better alleviate the financial burden on patients, efforts should be made to expand the breadth of the current protection system. For example, the inclusion of eligible rare disease medicines in the NRDL should be further promoted. Given that the conventional evaluation process for inclusion of new medicines in the NRDL is often time-consuming and complex, targeted adjustments and simplification of the reimbursement assessment procedures for rare disease medicines should be considered. Meanwhile, the applicable indications for reimbursed medicines should be further expanded to cover the relevant rare disease conditions that are not already included.

The experiences of other countries may provide useful reference regarding access to reimbursement for high-cost rare disease medicines. For example, Germany has adopted an approach in which some high-value medicines are initially included in reimbursement schemes based on relatively less restrictive criteria, before the collection of clinical evidence. After a trial period, the benefits of these medicines are then reassessed based on the accumulated evidence, and decisions are made regarding their formal inclusion in reimbursement systems and adjustments to the relevant levels of reimbursement (33). It should be noted that such mechanisms represent potential directions for institutional improvement based on the experiences of other countries, but their applicability in China requires further assessment, given the operational context of the Chinese health insurance system.

In addition, given the current financial sustainability pressures faced by China”s health insurance systems, the inclusion of high-cost rare disease medicines requires a balance between meeting patients’ treatment needs and controlling fund expenditure risks. Thus, in the course of expanding medicine coverage, risk-sharing mechanisms could be explored in combination with existing price negotiation and volume-based procurement mechanisms to mitigate potential payment risks. For example, some countries have already adopted financial risk-sharing mechanisms by negotiating payment caps or patient number limits with pharmaceutical suppliers. They have also implemented outcome-based risk-sharing mechanisms, under which payment responsibilities are adjusted using discounts, price reductions, or refunds according to the actual effectiveness of medicines. In the context of China’s health insurance payment system, future policy development could explore payment approaches combining Diagnosis-Related Groups (DRG) / Diagnosis-Intervention Packet (DIP) -based payment and special case negotiation mechanisms. For instance, high-cost rare disease medicines could be considered for negotiation-based payment on a case-by-case basis, while other related medical service costs could continue to be reimbursed through DRG/DIP-based payment mechanisms (34).

Meanwhile, considering the insufficient coverage for supportive medicines identified in this study, future policies could incorporate integrated reimbursement arrangements that include both therapeutic medicines and supportive medicines for rare diseases, thereby improving the ability of existing protection systems to address patients’ actual healthcare needs.

Furthermore, based on the finding that non-pharmaceutical direct medical expenses remain insufficiently covered, regions could, where appropriate, consider incorporating rare disease patients into catastrophic illness insurance programs to further strengthen financial protection for them.

2 Expanding the depth of reimbursement coverage

A tailored reimbursement approach should be adopted to address the specific challenges faced by patients with rare diseases. For example, to address the low reimbursement rates associated with out-of-area healthcare use, patients diagnosed with conditions that are included in the national rare disease lists could be automatically registered through the medical insurance system by designated healthcare institutions at the time of diagnosis (i.e., “diagnosis-triggered registration”). This would allow these patients to receive the same reimbursement rates for out-of-area care as are available in their home region. For children and for adults who are unable to work because of their condition, who are covered only by the resident insurance scheme, a dedicated reimbursement pathway could be established to provide outpatient reimbursement rates comparable to those available under the employee insurance scheme.

Even if reimbursement rates are improved, patients with rare diseases may still face substantial financial burdens. However, Medical Assistance could play a greater role in providing supplementary financial protection. For patients experiencing illness-induced poverty, or those already classified as low-income or in “five-guarantee” households (a vulnerable group covered by China’s social assistance system), a cap on out-of-pocket expenses could be established to further reduce their financial burden.

3 Promoting equity through the harmonization of coverage levels

To ensure that patients have equitable access to financial protection across regions, national-level policies should be considered for establishing and implementing phased targets, gradually harmonizing the approaches and standards for covering rare disease-related medical expenses across regions, and reducing regional disparities in the level of protection.

In the short term, priority should be given to two areas of standardization: (a) establishment of a unified outpatient coverage list for chronic and special diseases based on the National Catalogue of Rare Diseases, while standardizing both the scope of diseases that are covered and the reimbursement levels; and (b) harmonizing special payment policies across regions under CII by introducing a tiered reimbursement mechanism and establishing national benchmarks for deductibles and reimbursement ceilings for rare diseases, while allowing local authorities some degree of limited flexibility within a nationally defined framework.

In the long term, the objective should be to establish a national fund to support rare disease patients. Evidence drawn from comprehensive evaluations of pilot initiatives in provinces such as Zhejiang and Jiangsu could be used to inform the gradual development of a nationally unified reimbursement framework, ultimately enabling management and reimbursement of rare disease medical expenses to be coordinated on a national scale.

##### 4.1.1.2 Strengthening market protection

1 Reducing barriers to market-based coverage

The findings of this study indicate that some patients with rare diseases have difficulty obtaining effective protection through market-based insurance channels due to factors such as pre-existing condition and genetic disease exclusion clauses in commercial insurance products. Therefore, efforts should be made to explore new ways to design and operate commercial insurance products, so as to improve the accessibility of insurance protection for patients with rare diseases.

Specifically, commercial insurers could gradually optimize their coverage design for rare diseases based on product operation conditions, reduce eligibility restrictions as appropriate, and expand insurance coverage for patients with rare diseases. Regarding exclusion of those with pre-existing conditions, some countries have explored policy interventions to improve insurance accessibility for high-risk populations. For example, relevant policies in the United States have limited insurers’ use of pre-existing condition exclusions so as to allow individuals with pre-existing conditions, including those with rare diseases, to obtain insurance coverage (35). These international experiences may provide useful references for improving the protection mechanisms of commercial insurance for patients with rare diseases in China.

Expanding coverage to include patients with rare diseases may increase the claim risks of insurance products. Future policies could thus explore more flexible risk-sharing mechanisms based on actuarial assessments. For example, approaches such as reasonable premium adjustments and optimization of benefit designs could be considered as ways to improve financial protection for patients with rare diseases while maintaining the sustainability of commercial insurance products.

Such optimization approaches could also be gradually explored for genetic disease-related exclusion clauses, according to the operational conditions of different commercial insurance products. In this fashion, the possibility of patients with rare diseases obtaining market-based protection could be improved while still maintaining controllable insurance risks.

2 Developing a multi-tiered insurance product system

A multi-tiered insurance product system could be established, consisting of basic, enhanced, and premium coverage tiers, with each tier designed to meet the needs of different patient groups. For example, the basic tier could primarily serve individuals who have not been diagnosed with a rare disease but have a family history or genetic predisposition. The enhanced tier could serve patients who have been diagnosed with rare diseases, but whose conditions are relatively stable and whose medical expenses remain manageable. The premium tier could be designed for patients with substantially higher medical expenses and more intensive treatment needs.

The specific eligibility criteria for these tiers could be determined by insurers based on actuarial assessments and risk stratification principles. Premiums, benefit levels, and coverage scope could then be differentiated across tiers according to actuarial risk assessments, thereby providing more tailored protection for patients with varying levels of need.

Specifically, the basic tier would maintain the existing scope of coverage. The enhanced tier would expand coverage to include outpatient medical expenses and limited specialty drug benefits, in addition to those provided under the basic tier. The enhanced tier could also be implemented through an optional supplementary coverage package that would allow enrollees who require outpatient coverage to obtain additional benefits through voluntary enrollment.

The premium tier would expand specialty drug coverage further and include select indirect medical expenses (e.g., special medical food expenses) in addition to the benefits provided under the basic and enhanced tiers. The specialty drug formulary could also be aligned with the Category C Pharmaceutical Catalog introduced by the NHSA. This catalog is intended to cover highly innovative drugs with substantial clinical value that cannot yet be included in the NRDL because of the latter’s focus on basic healthcare protection (36). Rare disease drugs are particularly well suited to this policy objective.

On December 7, 2025, the NHSA issued the first edition of the Commercial Health Insurance Innovative Drug List, which includes several rare disease drugs. This list provides a national reference for Huiminbao drug coverage, and thus it could facilitate greater consistency across regional Huiminbao products. As the Category C Pharmaceutical Catalog expands, the scope of specialty drug coverage under Huiminbao could be expanded accordingly.

3 Strengthening operational risk management mechanisms

To enhance product stability and support longer-term coverage, operational risk management mechanisms should be strengthened, with a particular focus on actuarial sustainability. Insurance companies could be encouraged to adopt the following measures to improve actuarial sustainability.

First, actuarial capacity should be strengthened to support accurate premium pricing and claims management. This could be accomplished by encouraging insurers to collaborate with BMI authorities, social organizations, and research institutions to obtain key data on disease incidence, drug utilization, and medical expenses that could inform more accurate estimation of the current and projected rare disease population in each region. Insurers could thereby develop risk-adjusted premium structures and reimbursement policies that more closely reflect actual risk levels.

Second, to prevent adverse selection from intensifying and leading to a death spiral, the participation of healthy individuals should be continuously expanded. Specific strategies to improve enrollee retention and strengthen the risk pool include offering additional benefits to claim-free policyholders, allowing personal medical insurance accounts to be used to pay premiums for family members, and creating incentives for continuous enrollment through measures such as progressively lower deductibles for renewing policyholders.

Finally, subject to actuarial sustainability, coverage periods could be gradually extended by transitioning from annual policies to renewable multi-year products (e.g., two- or five-year terms) as a way to enhance coverage continuity and product stability.

##### 4.1.1.3 Strengthening social organization protection

1 Strengthening the capacity to mobilize charitable resources

The limited level of financial support provided by rare disease social organizations’ assistance programs may be due to their insufficient fundraising capacity. Strengthening fundraising capacity could thus potentially enable these organizations to attract more donations and, in turn, provide greater assistance to patients with rare diseases. There are a number of possible strategies for doing so.

First, their credibility and public visibility should be enhanced. This could be achieved by showcasing successful patient assistance programs, leveraging the mainstream media, strengthening their organizational branding, and establishing partnerships with hospitals, leading universities, and research institutions. These efforts could increase public awareness and trust, thereby expanding funding opportunities.

Second, funding sources should be diversified. In addition to actively seeking financial support from the central and local governments, social organizations should also be encouraged to collaborate with digital platforms to develop charity-linked consumption models (e.g., donation points through payment applications or charitable sections on e-commerce platforms) that could reduce barriers to public participation. Drawing on experience from Japan, donation mechanisms could also be integrated into vending machine sales, with a portion of proceeds directed toward supporting patients with rare diseases. International fundraising opportunities should also be explored through simplified cross-border donation procedures and partnerships with overseas charitable foundations to expand financial support for rare disease initiatives.

Finally, the government should strengthen tax incentive policies for charitable giving. By expanding tax deductions for charitable donations, enterprises and individuals could be encouraged to allocate more resources to rare disease social organizations.

2 Establishing a sustainable project operation model

The limited sustainability of charitable programs may partly result from inadequate financial management capacity among rare disease social organizations, which may restrict them to implementing only short-term programs as a cost-control measure. These organizations should enhance the sustainability of their assistance programs by preserving and increasing the value of their assets and optimizing the allocation of funds, thereby establishing a model for the continuous operation of such programs.

First, the government should encourage and support rare disease social organizations to undertake prudent and well-regulated investment activities that can generate sustainable financial returns, thereby enhancing their long-term financial sustainability.

Second, charitable resources should be allocated more efficiently to maximize the impact of available funding. To facilitate targeted assistance, standardized assessment tools could be developed based on the national rare disease lists. For example, a “Criticality Scoring Card” could be established, incorporating indicators such as clinical urgency and household income to determine assistance priority. These tools could be used to prioritize aid for the most vulnerable, such as households experiencing financial hardship and patients for whom interruption of treatment could result in severe health consequences.

3 Building a collaboration network among social organizations

It is recommended that a collaborative network be established by connecting different social organizations to facilitate information exchange and resource sharing among them and to develop coordinated assistance mechanisms. Such a network could help reduce disparities among organizations serving different rare disease groups in terms of their resource integration capacity and enable more resources to be directed toward diseases affecting particularly small patient populations.

Hub organizations for rare disease support could be established as well. Referring to the *Administrative Measures for the Evaluation of Social Organizations* issued by the Ministry of Civil Affairs, highly rated rare disease social organizations could be selected to serve as coordination hubs based on their demonstrated capacity for resource coordination and information dissemination. Local authorities could adjust the specific selection criteria according to the development status of existing rare disease social organizations within their jurisdictions.

These hub organizations could then serve a coordinating role by establishing digital volunteer service platforms for rare diseases and creating mechanisms for joint resource sharing and capacity building by rare disease charities, especially smaller disease-specific organizations. In addition, a partnership model could be implemented under which larger organizations would share their fundraising expertise with smaller organizations and collaborate with them in designing and evaluating charitable assistance programs for diseases that remain underserved by existing initiatives.

#### 4.1.2 Establishing a collaborative innovation mechanism among protection channels

Welfare pluralism holds that the protection of medical expenses for patients with rare diseases cannot rely solely on a single actor, but instead requires collaboration among multiple stakeholders. Therefore, in addition to improving the effectiveness of each of the three protection channels, a collaborative mechanism for developing new innovations to protect the medical expenses of patients with rare diseases could be developed. This mechanism would consist of three components: the functional positioning of each entity, coverage scope, and the reimbursement process.

##### 4.1.2.1 Functional positioning: clarifying expense-sharing responsibilities

**Figure 4:**
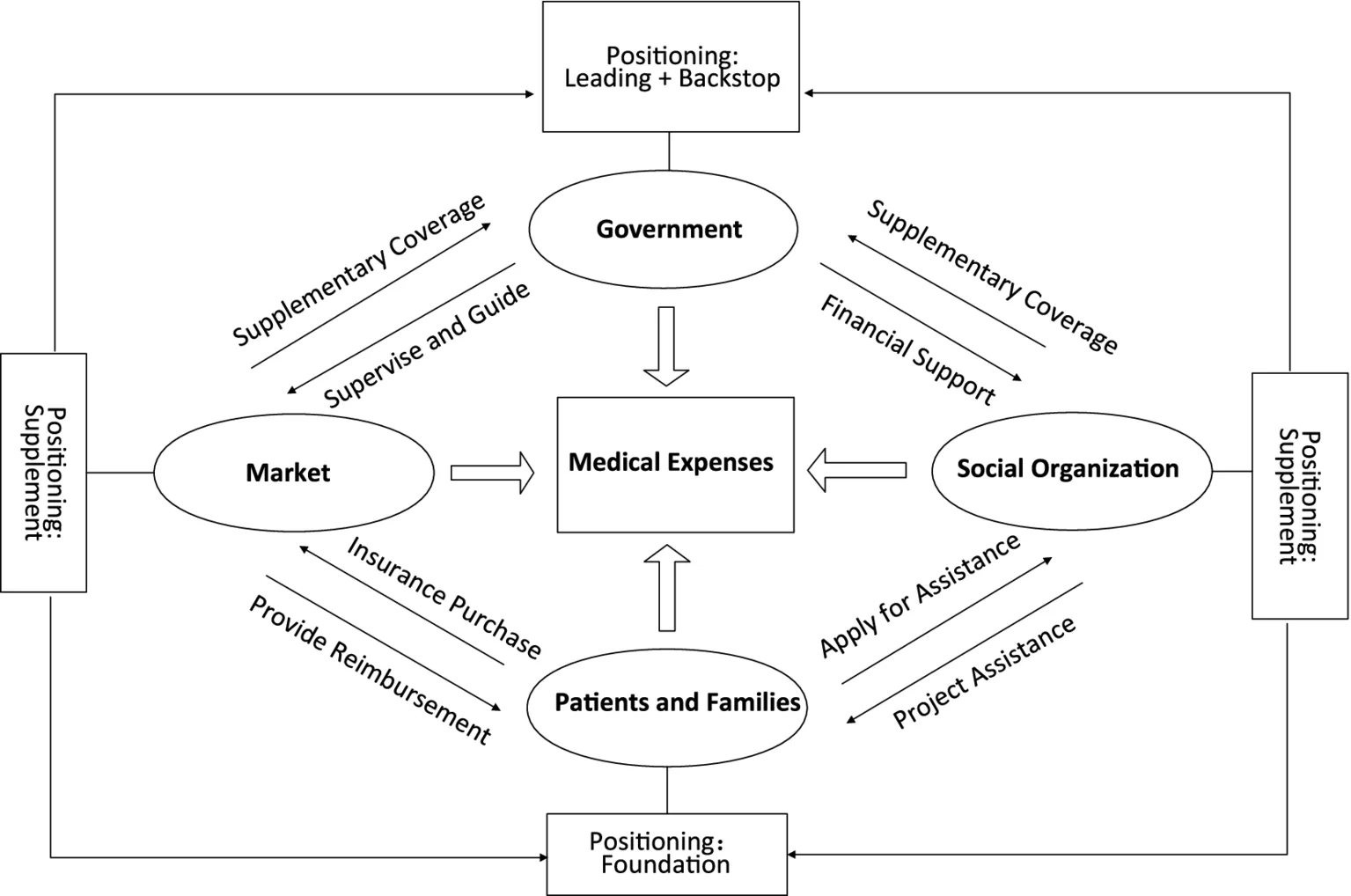
Expense-sharing responsibilities of each entity.

In addition to the three major entities (i.e., the government, the market, and social organizations), patients and their families remain important contributors to the financing of medical expenses associated with rare diseases. Although all four entities share the common objective of reducing the financial burden on patients with rare diseases and their families, each should perform a distinct function and assume clearly defined responsibilities while coordinating closely with the others. If collaboration can be made more effective, then a more comprehensive social protection safety net can be established for patients with rare diseases, thereby reducing their overall financial burden (see Figure 4).

First, the government should serve as the lead entity in the rare disease medical expense protection system and assume ultimate responsibility for protecting patients against catastrophic financial risk. From a social perspective, protection against medical expenses associated with rare diseases is a matter of social welfare, and healthcare protection is a public good that requires government leadership. From a legal perspective, the government is responsible for safeguarding patients’ rights to life and health and for preventing illness-induced poverty among patients with rare diseases.

In practice, the government’s relationship with the other three entities can be described as follows: it provides regulatory oversight and policy guidance to the market, financial and policy support to social organizations, and social insurance protection for patients and their families.

Both the market and social organizations can play supplementary roles in rare disease medical expense protection, with distinct functions. The market should capitalize on its capacity for innovation by offering diverse and flexible insurance products, thereby broadening available protection options for patients. Social organizations, meanwhile, primarily serve to address gaps that remain unfilled by government and market mechanisms. Given that their welfare provision is characterized by voluntary participation and limited sustainability, positioning them as complementary entities, rather than as sufficient in and of themselves, is both appropriate and necessary.

In practice, both the market and social organizations can complement the government’s role in rare disease medical expense protection: the market by providing commercial insurance coverage and reimbursement for patients, and social organizations by delivering program-based financial assistance and support services.

Finally, patients and their families should serve as the ultimate foundation of the protection system. As the most fundamental social institution, the family plays a key role in providing support in the face of life-course risks, including illness, disability, aging, and death. Families therefore bear a share of responsibility for protection, and their financial contributions constitute an important component of the overall protection system.

However, families’ capacity to absorb financial risk is limited, and thus an excessive burden should not be shifted onto patient households. For example, the proportion of medical expenses borne by patients should ideally remain below the threshold for catastrophic health expenditure, defined as out-of-pocket health expenditures exceeding 40% of a household’s capacity to pay (37).

Family contributions should thus remain within a manageable range, including paying BMI premiums, purchasing commercial insurance products, and seeking assistance from social organizations.

##### 4.1.2.2 Coverage scope: defining covered expenses and target patient populations

To improve the efficiency of the collaborative mechanism, the types of expenses covered and the target populations served by government, market, and social organization channels should be clearly defined. Because patients and their families contribute across all categories of expenses, they are not included in this section.

With regard to expense allocation, BMI and CII’s institutional roles imply that they should primarily cover direct medical expenses included within the “Three Catalogues” of China’s National Medical Insurance system. Huiminbao should primarily cover direct medical expenses not included in the “Three Catalogues”; it could also be expanded to include select indirect medical expenses, such as special medical food expenses and transportation expenses associated with out-of-area healthcare utilization. Social organization assistance programs, in addition to covering assistive device costs, could broaden their focus to include indirect medical expenses such as accommodation and travel expenses incurred during treatment.

From the perspective of target patient populations, patients with rare diseases can be divided into three categories. The first category consists of patients whose treatments are currently covered by drugs included in the NRDL. The second category comprises patients whose diseases are included in China’s rare disease lists, even though their treatments are not covered by the NRDL. The third category includes patients whose diseases are not included in China’s rare disease lists.

**Figure 5:**
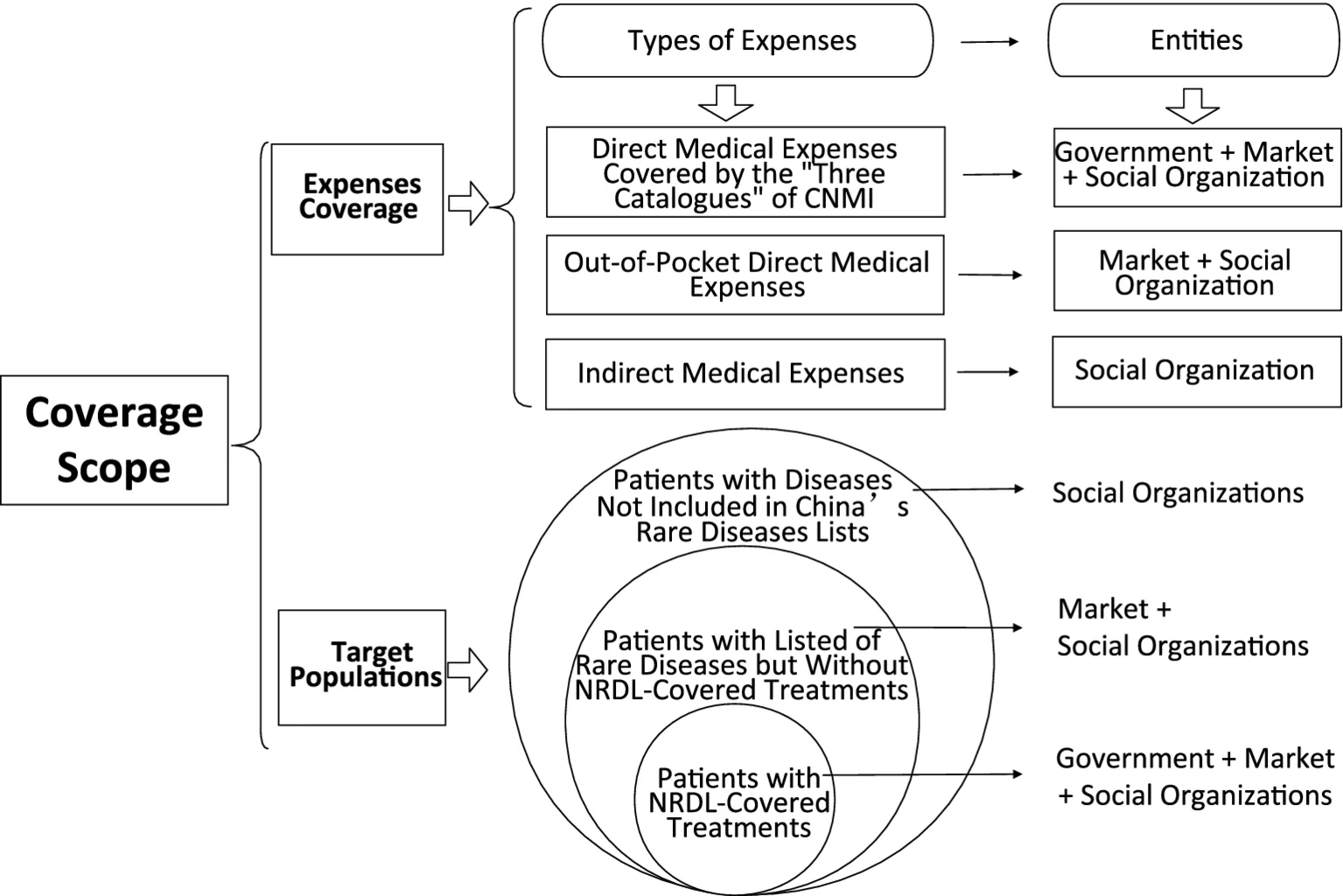
Types of expenses covered and focus of target patient populations by each entity.

At present, the first category receives the greatest level of policy attention and protection, while the third category typically faces more severe protection gaps and requires greater policy attention and support. To improve the effectiveness of the overall protection system, the primary target populations of each protection channel should be clearly defined. Specifically, government protection should primarily focus on the first category; the market should primarily target the second category while providing supplementary coverage for the first; and social organizations should prioritize the third category while offering complementary support to the first and second (see Figure 5).

##### 4.1.2.3 Reimbursement process: establishing a collaborative reimbursement mechanism

To address the problems of cumbersome procedures and fragmented reimbursement across multiple channels, a coordinated multi-stakeholder reimbursement process should be established, incorporating a one-stop reimbursement platform. This approach would strengthen interconnections among the participating stakeholders, improve the efficiency of financial protection, and provide patients with rare diseases with more convenient access to reimbursement.

**Figure 6:**
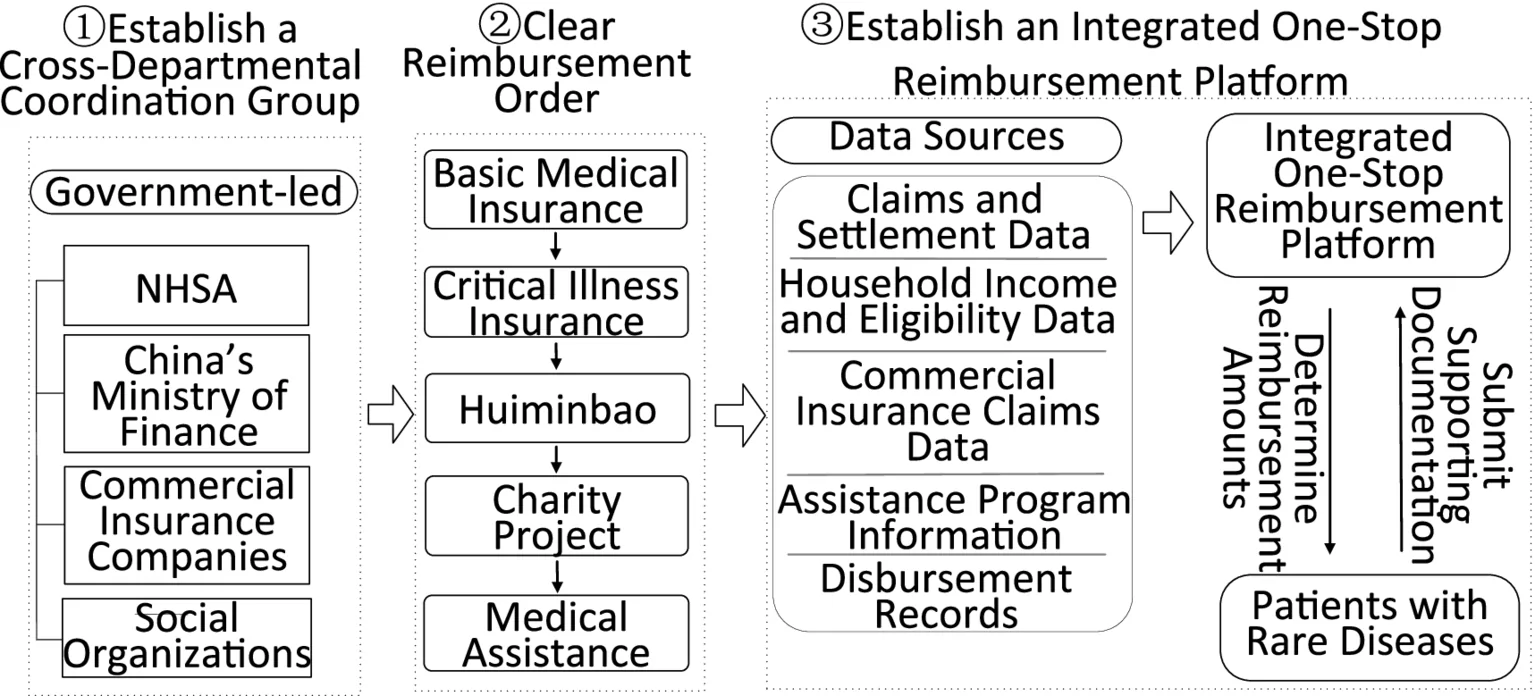
Establishing a collaborative reimbursement mechanism.

As the lead entity, the government should establish a cross-sector coordination body to facilitate the development of a coordinated reimbursement mechanism for rare disease medical expenses. This body would include the National Healthcare Security Administration, the Ministry of Finance of the People’s Republic of China, commercial insurance companies, and social organizations (see Figure 6).

The reimbursement order across protection channels should be clearly defined as well. Based on the functional positioning of the entities described above, BMI and CII should serve as the primary reimbursement channels, followed by Huiminbao, and then charitable assistance programs, with MA functioning as the payer of last resort.

During implementation, specialized coordination mechanisms should be established to improve administrative efficiency and service delivery by addressing issues such as disputes over payment responsibility, temporary funding shortages, and commercial insurance claim denials.

Finally, all relevant entities should collaborate to establish a one-stop reimbursement platform that consolidates data from BMI, commercial insurance, and charitable assistance programs. This platform would enable real-time verification of patient eligibility information and digitization of medical reimbursement receipts, thereby reducing administrative review burdens across entities and potentially simplifying documentation requirements.

Specifically, a dynamic rare disease patient registry (38) should be established, incorporating information such as duration of local residency and insurance enrollment status. The one-stop reimbursement platform could then be developed from this registry. The platform should integrate claims and settlement data from BMI authorities, household income and eligibility verification data from civil affairs departments, claims data from commercial insurers, and assistance program information and disbursement records from social organizations.

Implementation of this plan could proceed in phases. First, one-stop settlement bridging BMI and Huiminbao should be introduced, facilitating data exchange between BMI authorities and commercial insurers, while civil affairs departments should synchronize eligibility verification for low-income and extremely disadvantaged households. Thereafter, social organization assistance programs should be incorporated into the one-stop settlement system as well.

In addition, a Unified Supporting Documentation List for Rare Disease Reimbursement should be developed, such that patients could submit supporting documentation through a single application process to obtain reimbursement from multiple entities, thereby reducing duplicate documentation requirements and administrative review procedures and improving reimbursement efficiency.

At the same time, privacy protection and barriers to cross-departmental data sharing must be carefully addressed. According to the Administrative Data Sharing Regulations, government data sharing must be conducted while ensuring data security and confidentiality. Therefore, the scope and mechanisms of interdepartmental data sharing should be clearly defined, and appropriate encryption and data protection technologies should be used to safeguard personal information and prevent unauthorized disclosure of patient data.

### 4.2 Key findings

The results from the questionnaire survey indicate that, the patients who were reached through patient organizations and related networks face a substantial financial burden of rare disease-related medical expenses. This burden includes not only direct medical expenses, such as high drug and surgical costs, but also indirect medical expenses, such as round-trip transportation, accommodation outside medical institutions while traveling, and social and interpersonal expenses. Further analysis of the protection provided by the three protection channels for rare disease-related medical expenses revealed that coverage is limited and that patients’ out-of-pocket expenses are high. The incomes of patients and their families are insufficient to cover these high medical expenses, leaving many households heavily in debt.

The findings from the subsequent semi-structured interviews indicate that the three major protection channels have two main limitations in covering the medical expenses of the patients with rare diseases contacted in this study. Each of the three channels offers limited protection effectiveness. Specifically, the government channel has limited breadth of coverage, limited depth of coverage, and substantial inequities. The market channel is constrained by restrictive eligibility requirements, limitations in coverage scope, and instability of coverage. The social organization channel faces difficulties in addressing patient needs because it provides insufficient financial assistance, has support resources with limited long-term sustainability, and has uneven distribution of support resources across disease categories. Moreover, coordination among the three protection channels is limited, mainly in terms of overlapping coverage across entities and the absence of standardized cross-entity coordination procedures.

In light of these deficiencies, China’s current protection approaches for rare disease medical expenses should be improved in two ways: by enhancing the effectiveness of current protection channels, and by establishing a collaborative innovation mechanism among the protection channels. Although the international literature has examined approaches to financial protection for patients with rare diseases, most studies thus far have focused on individual actors, such as the government (4, 39) or social organizations (6, 40). Given the high and diverse medical expenses faced by patients with rare diseases, no single actor is likely to be able to provide comprehensive protection. Collaboration among multiple stakeholders is thus essential for improving coverage of these expenses and alleviating the financial burden on patients. Although some scholars have proposed multi-stakeholder collaboration at the macro level to alleviate the medical financial burden faced by patients with rare diseases (10), these approaches have generally been overly broad and underdeveloped, with limited attention to how different stakeholders can protect medical expenses at the operational level. In contrast, this study examines the distinctive functional positioning, scope of protection, and reimbursement processes of the government, market, and social organizations, and develops a more detailed collaborative innovation mechanism that connects the different protection channels.

### 4.3 Limitations

This study has several limitations worth noting. First, during questionnaire distribution, the questionnaires were distributed with the assistance of rare disease patient organizations; this may have systematically excluded marginalized patients who had not joined such organizations and thereby introduced selection bias into the survey results. During questionnaire completion, because patients’ medical expenses were based on self-reporting, the results may have been subject to recall bias, leading to deviations from their actual medical expenses. In terms of sample representativeness, although the study sample covered most provinces and cities in China, the overall sample size was relatively small because of limited human and financial resources, which may have limited its representativeness. Therefore, the medical expense burden for patients with rare diseases in China as estimated in this study may deviate to some extent from the actual situation. This bias may have influenced the results in opposite directions, as patients with more severe conditions, higher medical expenses, or a greater tendency to actively seek support may have been overrepresented, whereas those with limited connections to patient organizations, restricted digital access, or fewer social resources may have been underrepresented. However, the net direction and magnitude of any such bias are difficult to determine. Furthermore, because the questionnaire primarily focused on diseases included in China’s rare disease lists, even though a small number of diseases outside these lists were also included, the findings may present an overly optimistic picture of patients’ medical expense burden, as listed rare diseases are generally more likely to receive medical insurance reimbursement.

The interview component of this study has some limitations as well. The use of snowball sampling may have introduced selection bias and limited the diversity of interview perspectives. In addition, the use of proxy respondents may increase the risk of information bias, as their responses may not fully reflect the patients’ own experiences.

## 5 Conclusion

The findings of this study show that many patients with rare diseases who were contacted through patient organizations and related networks face a substantial financial burden associated with their medical expenses. The protection channels provided by the government, market, and social organizations have considerable room for improvement. Efforts should therefore be made, both to enhance the effectiveness of the existing protection channels and to develop a collaborative innovation mechanism bridging these channels to alleviate the medical financial burden on patients with rare diseases. Specifically, with regard to enhancing the effectiveness of current protection channels, the government’s role as the lead entity should be strengthened, the market channel’s protective function should be reinforced, and the support capacity of social organizations should be improved. With regard to establishing a collaborative innovation mechanism among the protection channels, the responsibilities of multiple entities should be clarified, the types of expenses covered by each entity and the target patient populations should be defined, and a collaborative reimbursement mechanism should be established.

## Data Availability

The original contributions presented in the study are included in the article/Supplementary material, further inquiries can be directed to the corresponding author.
