# Supplementary material for: Research on the innovation of medical expenses protection mechanism for rare disease patients in China
Source: Front Public Health. 2026 Sep 4;14:1847004. doi: 10.3389/fpubh.2026.1847004 (PMC13585904; doi:10.3389/fpubh.2026.1847004)
Supplement: Supplementary file 1 [file Data_Sheet_1.PDF]

# Interview Guide

## I. Basic Information of Patients with Rare Diseases

1. What is your age?

2. What is your level of educational attainment?

3. What rare disease do you have, and when did symptoms first appear?

4. What is your employment status and income level?

5. How many family members do you have, and what is your family's annual household income?

## II. Participation in Medical Insurance among Patients with Rare Diseases

1. Are you enrolled in any of the following: Basic Medical Insurance for Urban Employees / Basic Medical Insurance for Urban and Rural Residents / Critical Illness Insurance (CII) / other medical insurance?

2. Do you have any commercial health insurance or inclusive commercial health insurance (Huiminbao)?

3. Have you ever received Medical Assistance (MA)?

4. Have you ever received assistance from social organizations?

## III. Medical Expense Burden and Reimbursement among Patients with Rare Diseases

1. Is there a treatment available for your rare disease? What is the treatment?

2. How do you obtain treatment drugs? Have you encountered difficulties during the treatment process, and if so, what are they?

3. What are your average total medical expenses for treating the rare disease, and how are the various expenses itemized?

4. Do you or your family experience financial burden in covering medical expenses? What range of rare disease medical expenses can you afford?

5. If you are covered by Basic Medical Insurance (BMI), what is the reimbursement status under BMI and CII? Have you encountered difficulties during the reimbursement process?

6. If you have received MA, what is the reimbursement status? Have you encountered difficulties during the reimbursement process?

7. If you have purchased commercial health insurance or Huiminbao provided by the market, what is the reimbursement status? Have you encountered difficulties during the reimbursement process?

8. If you have received assistance from social organizations, what is the reimbursement status? Have you encountered difficulties during the reimbursement process?

**IV. Suggestions for the Rare Disease Medical Expense Protection System**

1. What proportion of medical expense reimbursement would you like to receive? Which category of expenses would you most like to see reimbursed?

2. Do you believe that the current regional rare disease medical expense protection system has shortcomings? If so, what are they?
